# Vitamin D Supplementation in Orthopedic Trauma: Influence on Immune Modulation, Fracture Healing, and Infection Outcomes

**DOI:** 10.7759/cureus.97842

**Published:** 2025-11-26

**Authors:** Gaurav Jha, Roopkaran Dhanjal, Ameer Hamza

**Affiliations:** 1 Trauma and Orthopedics, Leicester Royal Infirmary, Leicester, GBR; 2 Orthopedics, Guy's and St Thomas' NHS Foundation Trust, London, GBR; 3 Emergency Medicine, Barking, Havering and Redbridge University Hospitals NHS Trust, London, GBR; 4 Trauma and Orthopedics, University Hospitals of Leicester NHS Trust, Leicester, GBR

**Keywords:** fracture healing factors, nonunion, orthopedic infection, periprosthetic joint infection, vitamin d deficiency

## Abstract

Vitamin D deficiency is a common and correctable problem in orthopedic patients, increasingly recognized for its impact on infection risk, delayed fracture healing, and poor surgical outcomes. Beyond its role in maintaining calcium and phosphate balance, vitamin D supports immune and bone health by promoting antimicrobial peptide production, macrophage activity, and osteoblast function, all of which are essential for infection control and bone repair. This review draws on studies in adult orthopedic populations that assessed vitamin D status in relation to surgical site and periprosthetic infections, fracture healing, nonunion, and fracture prevention. Across these studies, deficiency below 30 ng/mL was found in 43-90% of patients. This widespread deficiency was consistently linked to higher infection rates, with infected patients showing lower vitamin D levels at surgery. Postoperative levels decline by up to 40%, which can worsen existing insufficiency during the critical early recovery period when infection risk is highest. Observational and prospective data show that ongoing deficiency delays or prevents fracture union, with registry and cohort studies confirming a measurable rise in nonunion risk. Overall, the evidence supports vitamin D as a modifiable factor influencing orthopedic recovery. Routine screening and supplementation of at least 800 IU daily to maintain serum levels above 30 ng/mL are simple, safe, and inexpensive measures that can reduce infections, support bone healing, and lower fracture risk. Hospitals should incorporate vitamin D screening, correction, and maintenance into standard orthopedic care. This narrative review summarizes current evidence on vitamin D and orthopedic outcomes and provides practical guidance for its perioperative management.

## Introduction and background

Complications following orthopedic trauma and elective surgery, including surgical site infection, fracture-related infection, delayed union, and nonunion, represent leading causes of patient morbidity, prolonged rehabilitation, and substantial healthcare expenditure worldwide. Surgical site infection rates following trauma fixation range from 1% to 15%, depending on injury severity and soft tissue damage, while nonunion affects up to 10% of fractures despite advances in fixation techniques [1]. These complications extend hospital stays, necessitate revision procedures, and significantly impair functional recovery and quality of life. Outcomes reflect not only operative technique and mechanical stability but also host biology, including immunocompetence and nutritional status. Vitamin D has emerged as a critical, modifiable host factor that intersects both infection defense and osteogenesis [1,2].

Vitamin D functions as a steroid hormone with effects extending far beyond its classical role in calcium regulation and bone mineralization. Following cutaneous synthesis via ultraviolet B radiation or dietary intake, vitamin D undergoes hepatic 25-hydroxylation to produce 25-hydroxyvitamin D (25(OH)D), the principal circulating form and standard biomarker of vitamin D status [2]. Subsequent renal 1α-hydroxylation generates the biologically active metabolite 1,25-dihydroxyvitamin D (1,25(OH)₂D), which binds to vitamin D receptors (VDRs) expressed in nearly all tissue types, including immune cells, osteoblasts, and osteoclasts [2].

Within the immune system, active vitamin D promotes several protective mechanisms [3,4]. It activates macrophages (white blood cells that engulf bacteria), stimulates production of antimicrobial peptides called cathelicidin LL-37 and β-defensins (natural antibiotics produced by the body), enhances autophagy (a process that helps cells clear intracellular bacteria), and modulates regulatory T-cell function to balance immune responses. In skeletal tissue, vitamin D drives osteoblast differentiation, the process by which stem cells become bone-forming cells. It maintains calcium-phosphate balance necessary for bone mineralization and regulates the coupling between bone formation and bone breakdown through a signaling pathway called RANKL/OPG (a key regulatory mechanism that maintains bone strength). Additionally, vitamin D attenuates excessive inflammation that can impair healing [5,6].

Multiple studies document alarmingly high rates of vitamin D deficiency in orthopedic populations. Cross-sectional analyses of elective surgical cohorts reveal that 43-90% have serum 25(OH)D levels below 30 ng/mL (the threshold for sufficiency), with many frankly deficient below 20 ng/mL [7-9]. In one comprehensive study of 1,083 orthopedic inpatients, the mean level was only 17.1 ng/mL, with 86% insufficient and 60% frankly deficient [9]. Among trauma patients, deficiency rates are even higher, reaching 66% in some cohorts [7]. Sports medicine patients show a 52% deficiency [7]. Approximately half of the acute hip fracture admissions, a particularly vulnerable population, presented with vitamin D deficiency [10]. Elderly patients demonstrate the highest deficiency rates, with 86-90% of those aged ≥70 years classified as insufficient [9]. This epidemic reflects multiple converging factors: advanced age with diminished skin capacity to produce vitamin D, institutionalization with limited sunlight exposure, obesity (vitamin D gets sequestered in fat tissue), chronic medical conditions, geographic latitude limiting year-round sun exposure, seasonal variation with winter lows, and inadequate dietary intake [7-10].

Vitamin D deficiency directly impacts surgical outcomes through two critical pathways. On one hand, it impairs immune defense, increasing infection rates by up to sixfold, and on the other, it disrupts bone healing, raising nonunion risk nearly 30-fold [5,11]. These effects translate to longer hospital stays, higher revision surgery rates, and delayed return to function.

Given vitamin D’s pathophysiologic roles in immunity and osteogenesis, combined with near-universal deficiency in orthopedic patients, the potential clinical implications are profound. This narrative review synthesizes the current evidence base with a comprehensive understanding of vitamin D’s role in perioperative care, mechanistic insights into biological pathways, critical appraisal of existing evidence, and practical guidance for screening and supplementation protocols. Where evidence permits, we offer specific recommendations while highlighting knowledge gaps requiring future research.

## Review

Methods

A narrative literature review was conducted to examine the relationship between vitamin D status and orthopedic outcomes. We searched PubMed, Cochrane Central Register of Controlled Trials, and Embase. Search terms combined “vitamin D”, “25-hydroxyvitamin D”, “cholecalciferol”, and “hypovitaminosis D” with orthopedic-specific terms including “periprosthetic joint infection”, “surgical site infection”, “fracture healing”, “nonunion”, and “arthroplasty” using Boolean operators. The overview of the search methodology is detailed in Table 1.

**Table 1:** Overview of literature review methodology

Review parameter	Details
Review type	Narrative review
Databases searched	PubMed, Cochrane Central Register of Controlled Trials, and Embase
Population focus	Adult orthopedic patients (≥18 years)
Clinical context	Orthopedic surgery, trauma, and arthroplasty
Outcome domains	Surgical site and periprosthetic joint infection
	Fracture healing, nonunion, and delayed union
	Fracture prevention
Study types reviewed	Systematic reviews, randomized controlled trials, prospective and retrospective cohort studies, and case-control studies
Exclusions	Pediatric populations
	Animal or in vitro studies
	Conference abstracts and case reports
	Vitamin D analogues (calcitriol and alfacalcidol)
Synthesis approach	Thematic organization by infection, fracture healing, and prevention domains with emphasis on clinical applicability and mechanistic understanding

Vitamin D and orthopedic infection

The relationship between vitamin D status and orthopedic infection risk has been examined through multiple study designs, yielding remarkably consistent associations. Understanding how vitamin D deficiency increases infection risk is critical for improving surgical outcomes.

Evidence From Systematic Reviews and Case-Control Studies

Zargaran et al. conducted a systematic review of nine studies investigating vitamin D in orthopedic infection, concluding that deficiency was frequently associated with both periprosthetic joint infection (infection around joint replacements) and fracture-related infection [3]. However, substantial variation in study methods prevented definitive conclusions about causation.

Case-control studies provide more specific estimates of infection risk. One such study demonstrated substantially lower vitamin D levels in infected patients compared to uninfected controls (13.3 vs. 19.5 ng/mL) [4]. The authors proposed that impaired production of antimicrobial peptides and reduced macrophage activation in vitamin D-deficient states compromise the body’s defense mechanisms at the prosthesis-bone interface, where bacterial biofilm formation occurs [4].

In another case-control analysis of 205 patients by Zargaran et al., the infected group had a lower mean vitamin D than controls [11]. Low vitamin D status was associated with an adjusted infection odds increase of approximately sixfold, meaning deficient patients were six times more likely to develop infection. Seventy percent of infected patients were deficient, establishing vitamin D deficiency as a strong independent predictor of orthopedic infection.

Timing and Severity of Infection

Prospective matched-pair studies of knee or hip replacement cohorts show high overall deficiency rates reaching 87% [12]. Intriguingly, acute infections exhibited significantly lower mean 25(OH)D compared to chronic cases. This suggests that severe deficiency may predispose patients to early-onset, more aggressive infections, while those with borderline-deficient levels develop infections with longer latency periods.

Perioperative Vitamin D Fluctuations: A Critical Vulnerability Window

Beyond baseline associations, vitamin D levels demonstrate dynamic fluctuations during the perioperative period that may transiently worsen preexisting deficiency. This creates a window of vulnerability precisely when infection risk is highest. Patients undergoing elective knee replacement demonstrate a dramatic ~40% fall in vitamin D levels within 48 hours after surgery [13]. This acute drop coincided temporally with peaks in inflammatory markers CRP and IL-6, suggesting that surgical trauma triggers vitamin D consumption, redistribution, or sequestration during the acute inflammatory response.

Importantly, levels remained 20% suppressed at three months, indicating prolonged depletion [13]. These kinetics have critical implications during the vulnerable early postoperative window when most infections develop. In hip fracture patients, Fakler et al. found that low preoperative vitamin D combined with elevated CRP independently predicted postoperative medical complications but not one-year mortality [14]. This underscores the interplay between nutritional and inflammatory states in elderly trauma patients, where metabolic derangements amplify infection susceptibility.

Exceptions and Nuances in the Evidence

Not all studies demonstrate uniform associations, highlighting complexity and potential confounding. In a study conducted by Signori et al., overall deficiency prevalence was 79% across infection and control groups; patients with late periprosthetic infection paradoxically exhibited slightly higher means than aseptic loosening controls [15]. Critically, in a retrospective analysis of patients with osteoarticular infections, no association between baseline hypovitaminosis D and treatment success was found when patients received appropriate vitamin D repletion alongside antibiotics [16]. This observational study, while limited by retrospective design, suggests that deficiency represents a modifiable risk factor rather than an immutable predictor. When corrected promptly with supplementation, the adverse effects on immune function may be reversed, neutralizing infection risk.

Biological Mechanisms: How Vitamin D Protects Against Infection

**Figure 1:**
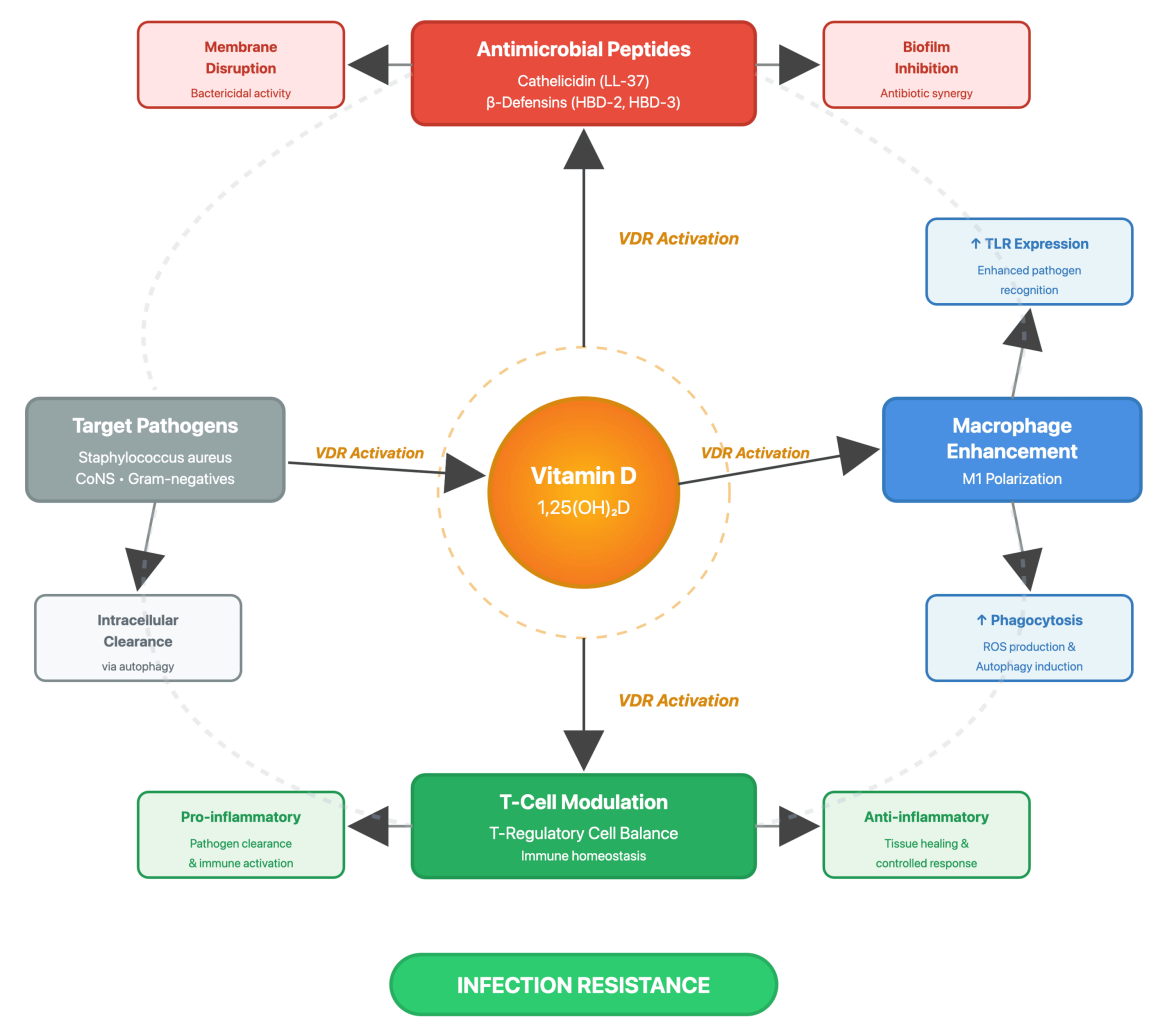
Schematic diagram of vitamin D immunological mechanisms in infection resistance CoNS, coagulase-negative staphylococci; HBD, human β-defensins; ROS, reactive oxygen species; TLR, Toll-like receptor; VDR, vitamin D receptor Adapted from references [3,4,12] Original illustration created by Gaurav Jha and Roopkaran Dhanjal

The biological plausibility linking vitamin D to infection resistance rests on well-established immunological mechanisms (Figure 1). These pathways explain how deficiency increases infection susceptibility and why supplementation may offer protection.

Antimicrobial peptide production: VDR activation in macrophages directly upregulates expression of cathelicidin (LL-37) and β-defensins (HBD-2 and HBD-3), antimicrobial peptides with broad-spectrum bactericidal activity against common orthopedic pathogens, including *Staphylococcus aureus*, coagulase-negative staphylococci, and Gram-negative organisms. These peptides disrupt bacterial membranes, inhibit biofilm formation (the protective coating bacteria form on implants), and work synergistically with conventional antibiotics [3,4].

Enhanced macrophage function: Beyond antimicrobial peptides, active vitamin D enhances macrophage function through multiple pathways [3,12]. It increases expression of Toll-like receptors (proteins that recognize bacterial components), improves pathogen recognition, promotes M1 polarization (a state that enhances bacteria-killing capacity), increases phagocytic capacity and production of reactive oxygen species that destroy bacteria, and induces autophagy to facilitate clearance of intracellular bacteria that evade extracellular immune responses [3,12].

Immune balance: At the T-cell level, vitamin D modulates regulatory T-cell function [4], balancing pro-inflammatory responses necessary for pathogen clearance against excessive inflammation that impairs tissue healing.

Clinical Implications for Infection Prevention

The aggregate evidence spanning systematic reviews, case-control studies, and cohort analyses establishes vitamin D deficiency as a reproducible correlate of orthopedic infection with biologically plausible mechanistic underpinnings [4]. While randomized trials specifically testing preoperative vitamin D supplementation to reduce infection rates remain limited, the consistency of observational associations combined with the safety and low cost of supplementation support proactive screening and correction. Given that deficiency increases infection risk approximately sixfold and that postoperative levels drop by 40% during the critical vulnerability window, ensuring adequate preoperative vitamin D status appears to be a rational, low-risk intervention to optimize surgical outcomes [11,13].

Vitamin D and fracture healing

Beyond its role in infection prevention, vitamin D deficiency also fundamentally disrupts the bone repair process itself. Fracture healing is a complex, multi-phase biological process involving inflammation, repair, and remodeling, all of which depend on systemic and local factors. Vitamin D is crucial in this cascade, regulating calcium-phosphate balance, osteoblast differentiation, and immune responses. Its deficiency has been consistently associated with delayed or failed union across different fracture types and populations.

Large-Scale Evidence: Registry and Cohort Studies

Zura et al. analyzed over 309,000 fractures across 18 anatomical sites, reporting a 4.9% overall nonunion rate, with vitamin D deficiency emerging as an independent predictor [1]. Although the relative risk increase appears small, the absolute population impact is significant, translating to thousands of potentially preventable nonunions annually. Evidence from another study conducted by Gorter et al. showed that 40% of 617 fracture patients were vitamin D deficient at presentation, and those who remained deficient had a 9.7% delayed union rate compared to 0.3% in non-deficient patients [5]. Importantly, patients whose deficiency was corrected had outcomes similar to those who were sufficient from the start [5], supporting a causal and reversible link. Brinker et al. also identified metabolic abnormalities in 84% of 37 patients with established nonunions, with vitamin D deficiency being most common (68%), and correcting these abnormalities led to fracture healing in several cases without surgery [17].

Vitamin D Dynamics During Fracture Healing

Serial measurement studies reveal that vitamin D levels decline during active bone repair. In a study by Ettehad et al., the results show that mean serum 25(OH)D levels dropped from 39.2 ng/mL at admission to 28.6 ng/mL by week 3 [18]. This was a significant drop of 26%. The most rapid decline occurred in the initial inflammatory phase, coinciding with hematoma formation and callus development. The proposed mechanisms include active consumption during mineralization, redistribution to fracture sites with increased 1α-hydroxylase activity, and sequestration during inflammatory responses [18]. These findings suggest that fracture patients experience heightened vitamin D demands during early healing. Patients who are already deficient at injury may become severely depleted during the critical healing period unless supplementation is provided.

Evidence From Randomized Controlled Trials

Randomized trials exploring vitamin D supplementation for fracture healing have shown mixed results but valuable insights. A double-blind randomized controlled trial study showed that 89% of long bone fractures resulted in patients who were vitamin D deficient (mean 16 ng/mL) [19]. Gorter et al.’s systematic review synthesized multiple small studies and concluded that, while individual results varied, overall trends supported a positive biological effect of vitamin D sufficiency on healing [6]. The review also highlighted methodological limitations, such as small sample sizes, inconsistent dosing, and heterogeneous outcomes, reinforcing the need for well-powered, standardized trials.

**Figure 2:**
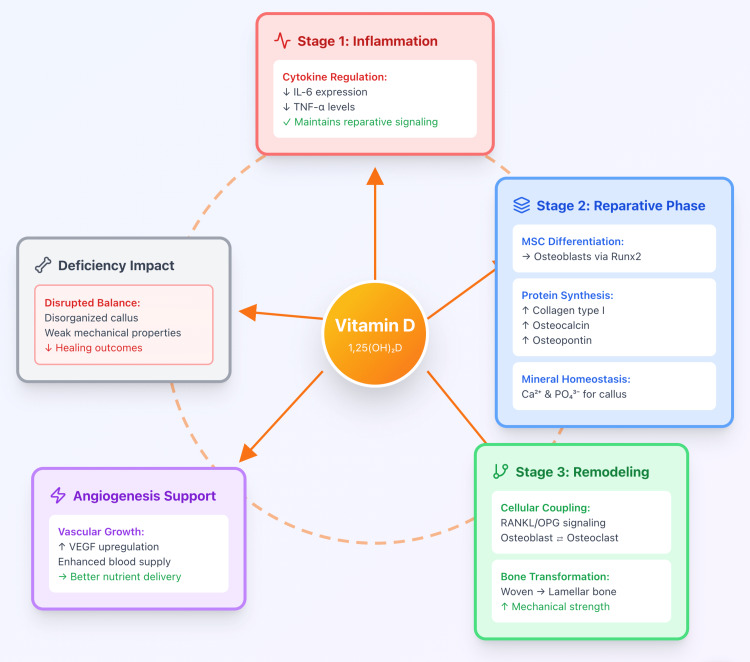
Mechanistic overview of immunological and cellular responses OPG, osteoprotegerin; RANKL, receptor activator of nuclear factor kappa-Β ligand; VEGF, vascular endothelial growth factor Adapted from references [5,6,17] Original illustration created by Gaurav Jha and Roopkaran Dhanjal

Biological studies help explain these clinical findings' pathophysiology (Figure 2). During the initial inflammatory phase, vitamin D regulates cytokine balance by reducing excessive production of pro-inflammatory molecules (IL-6 and TNF-α) while maintaining reparative signaling needed for healing initiation [6]. In the reparative phase, the active form of vitamin D (1,25(OH)₂D) drives mesenchymal stem cells to differentiate into osteoblasts via activation of Runx2 (a master regulatory protein for bone formation) [5,6]. It also enhances synthesis of key bone matrix proteins, including collagen type I, osteocalcin, and osteopontin. Additionally, vitamin D supports systemic calcium and phosphate homeostasis, ensuring sufficient mineral substrate for callus mineralization, the hardening of the initial soft callus into calcified bone [5,6].

During the remodeling phase, vitamin D coordinates the coupling between osteoblasts and osteoclasts through RANKL/OPG signaling, a regulatory pathway essential for converting the initial woven bone callus into strong, organized lamellar bone [1,5]. Deficiency disrupts this balance, leading to a disorganized and mechanically weak callus that is prone to failure. Moreover, vitamin D promotes angiogenesis through upregulation of vascular endothelial growth factor, improving vascular supply to the regenerating bone [17]. Adequate blood supply is critical for delivering oxygen, nutrients, and immune cells to the healing fracture site.

Clinical Recommendations for Fracture Management

Clinically, the evidence supports routine screening for vitamin D deficiency in fracture patients, especially those at high risk, like elderly individuals, those with limited sun exposure, obesity, or a history of nonunion. Early supplementation can be decisive, particularly within the first few weeks post-injury. In cases of refractory nonunion, metabolic profiling, including vitamin D, parathyroid hormone, calcium, phosphate, and thyroid function, should be routine. As demonstrated by Brinker et al., addressing these deficiencies can restore union without surgical intervention [17]. Incorporating vitamin D assessment and supplementation into fracture management protocols offers a low-cost, biologically sound strategy to enhance outcomes, reduce complications, and minimize healthcare burden.

Vitamin D deficiency prevalence and fracture prevention

Vitamin D deficiency is strikingly prevalent among orthopedic patients across all subspecialties and age groups. A study conducted by Maier et al. measured serum 25(OH)D in 1,083 orthopedic inpatients and reported mean levels of 17.1 ng/mL, with 86% insufficient (<30 ng/mL) and 60% frankly deficient (<20 ng/mL) [9]. In an elective surgery cohort group, 43% of the population group were found insufficient and 40% of these severely deficient, with the highest rates in trauma (66%) and sports medicine (52%) [7]. Male sex, higher BMI, and darker skin pigmentation independently predicted low levels, confirming that vitamin D deficiency extends beyond fragility fracture cohorts [7].

Seasonal and Geographic Variations

A multicenter follow-up by Maier et al. in 1,119 patients found 84% insufficiency and only 15% within target, with seasonal fluctuations showing a 39% decline from July to November [8]. Even at peak summer, mean concentrations remained suboptimal, highlighting that sunlight exposure alone is inadequate at temperate latitudes [8]. Among the frail elderly, Nurmi et al. reported similar findings in 223 acute hip fracture patients [10]. Over half were deficient, with marked winter deterioration prevalence reaching 67% in winter, in contrast to 33% in summer [10]. The preventive potential of vitamin D has been best demonstrated in randomized trials evaluating fracture risk. However, results have been inconsistent, largely due to differences in dosing, adherence, and baseline deficiency status.

Geography and season profoundly affect vitamin D status due to the angle-dependent penetration of ultraviolet B radiation. Above 35°N latitude, winter sunlight fails to induce adequate skin production of vitamin D from November through February [8,10]. Nurmi et al.’s Finnish cohort (61°N latitude) and Maier et al.’s German series (50°N latitude) both confirm that sunlight alone cannot maintain sufficiency in northern climates, particularly among elderly or immobile patients [8,10].

The landmark study by Chapuy et al. randomized 3,270 institutionalized elderly women to vitamin D plus calcium versus placebo [20]. The intervention group experienced a 43% reduction in hip fractures and 32% fewer nonvertebral fractures. These dramatic effects were attributed to severe baseline deficiency in the population, sufficient dosing (800 IU daily plus calcium), and strong adherence to the protocol. Conversely, the Women’s Health Initiative by Jackson et al. enrolled 36,282 postmenopausal women and found no significant reduction in hip fractures [21]. However, adherence was poor (59% at seven years), and the vitamin D dose was subtherapeutic. Among adherent women with calcium and vitamin D supplementation, hip fracture risk decreased 29%, suggesting the apparent null result stemmed from underdosing and low compliance.

Similarly, the RECORD trial randomized 5,292 adults ≥70 years with prior low-trauma fracture and found no fracture reduction in intention-to-treat analysis [22]. Adherence was again low (54.5% at two years), and many participants self-supplemented, diluting treatment effects. Per-protocol analysis showed a non-significant 29% hip fracture reduction among adherent patients, indicating efficacy may depend on consistent intake and baseline deficiency.

A participant-level meta-analysis by Bischoff-Ferrari et al. combined 11 double-blind trials to clarify this dose-response relationship [23]. While intention-to-treat analysis showed a non-significant 10% hip fracture reduction overall, stratification by actual vitamin D intake revealed that only the highest quartile (≥800 IU/day) achieved benefit with a 30% hip fracture reduction and 14% nonvertebral fracture reduction. These results confirm a threshold effect, showcasing that vitamin D must reach ≥800 IU/day, ideally in combination with calcium, to effectively prevent fractures.

Based on these data, target serum 25(OH)D levels in orthopedic patients should be ≥30 ng/mL (75 nmol/L), with an optimal range of 30-60 ng/mL [23]. Levels <20 ng/mL signify deficiency and require correction, while 20-30 ng/mL denotes insufficiency. Recommended repletion involves a loading phase of 50,000 IU cholecalciferol weekly for eight weeks (or a 100,000-300,000 IU single oral/intramuscular dose) followed by maintenance therapy of 800-2,000 IU daily [23]. Co-administration of 1,000 mg calcium daily improves mineralization, and levels should be rechecked after three months to ensure maintenance within the target range. Doses up to 2,000 IU/day are considered safe for the general population, while higher loading doses remain appropriate under supervision. Toxicity is rare and typically requires chronic intakes above 10,000 IU/day [8,10,23]. At temperate and northern latitudes, dietary and supplemental vitamin D should thus be regarded as essential components of orthopedic and fracture care rather than optional adjuncts.

Synthesis and clinical implications

When evidence across infection, fracture healing, and fracture prevention is considered collectively, a coherent pattern emerges identifying vitamin D as a modifiable determinant of orthopedic outcomes. The ubiquity of deficiency, ranging from 43% to 90% across orthopedic populations [7-10], combined with consistent associations with infection risk, delayed union (30-fold increase), and fracture risk reduction with supplementation (30%), supports a causal rather than correlative role. Mechanistic studies reinforce biological plausibility through VDR expression in osteoblasts, osteoclasts, and macrophages, promoting antimicrobial peptide synthesis, immune regulation, and bone matrix formation [6,17].

Interventional data, including the participant-level meta-analysis of 31,022 individuals, confirm a clear dose-response threshold with clear evidence that daily intake ≥800 IU reduces hip fractures by 30% and nonvertebral fractures by 14% [23,24]. Lower doses or single bolus regimens fail to sustain needed sufficiency, underscoring the necessity for continuous supplementation throughout the fracture healing period. Collectively, these findings establish a biologically and clinically coherent framework supporting vitamin D sufficiency as essential to optimize orthopedic recovery and long-term bone health.

Despite this evidence, vitamin D assessment and supplementation remain inconsistently adopted, and supplementation should be integrated into orthopedic care pathways. Evidence supports universal screening for fracture and arthroplasty patients, particularly those with high-risk profiles such as the elderly, obese, institutionalized, or those with darker skin [9,10]. Recommended regimens include a loading dose of 50,000 IU weekly for eight weeks or a single 100,000-300,000 IU dose followed by 800-2,000 IU daily maintenance with 1,000 mg calcium co-supplementation [24,25]. Levels should be rechecked after three months to maintain 30-60 ng/mL. Safety data remain reassuring. Doses up to 2,000 IU daily and loading regimens up to 300,000 IU are well tolerated, with hypercalcemia being rare and usually related to comorbid disorders [21]. Implementation studies demonstrate that structured screening and supplementation protocols can achieve >80% adherence and significantly improve patient outcomes [25].

Future research should focus on large-scale randomized controlled trials assessing vitamin D optimization for infection prevention and fracture healing, standardizing deficiency thresholds and outcome measures, and evaluating cost-effectiveness in real-world settings. Strengthening institutional protocols, clinician awareness, and adherence monitoring will be key to translating current evidence into improved patient outcomes.

## Conclusions

Vitamin D deficiency is widespread among orthopedic patients and has a clear impact on infection risk, fracture healing, and long-term bone health. Across trauma and elective settings, low vitamin D levels are consistently linked to higher complication rates, delayed recovery, and impaired bone regeneration. The biological rationale is well established through vitamin D’s role in immune modulation, osteoblast function, and calcium regulation. This mechanistic understanding reinforces the clinical relevance of maintaining adequate vitamin D levels throughout the perioperative period. Clinical translation of this evidence is straightforward: Routine screening and timely supplementation should be integrated into orthopedic care pathways, particularly for older adults, fracture patients, and those undergoing major reconstructive or revision procedures. With strong evidence of benefit, minimal cost, and excellent safety profiles, optimizing vitamin D status represents a simple yet powerful measure to enhance recovery, prevent complications, and improve outcomes throughout the orthopedic care pathway.
